# Group-based exercise and cognitive-physical training in older adults with self-reported cognitive complaints: The Multiple-Modality, Mind-Motor (M4) study protocol

**DOI:** 10.1186/s12877-016-0190-9

**Published:** 2016-01-16

**Authors:** Michael A. Gregory, Dawn P. Gill, Erin M. Shellington, Teresa Liu-Ambrose, Ryosuke Shigematsu, Guangyong Zou, Kevin Shoemaker, Adrian M. Owen, Vladimir Hachinski, Melanie Stuckey, Robert J. Petrella

**Affiliations:** Health and Rehabilitation Sciences, Western University, London, ON Canada; Lawson Health Research Institute, London, ON Canada; Department of Family Medicine, Western University, London, ON Canada; Department of Epidemiology, University of Washington, Seattle, WA USA; School of Kinesiology, Western University, London, ON Canada; Department of Physical Therapy, University of British Columbia, Vancouver, BC Canada; Djavad Mowafaghian Centre for Brain Health, Vancouver, BC Canada; Faculty of Education, Mie University, Tsu, Japan; Robarts Clinical Trials of Robarts Research Institute, Western University, London, ON Canada; Department of Epidemiology and Biostatistics, Western University, London, ON Canada; The Brain and Mind Institute, Western University, London, ON Canada; Department of Clinical Neurological Sciences, Western University, London, ON Canada; Canadian Centre for Activity and Aging, Faculty of Health Sciences, Western University, London, ON Canada; Centre for Studies in Family Medicine, Western Centre for Public Health and Family Medicine, Second Floor, Western University, 1465 Richmond St., London, ON N6G 2M1 Canada

**Keywords:** Multiple-modality, mind-motor, exercise, randomized controlled trial, older adults, cognitive complaints, cognition, mobility, vascular health

## Abstract

**Background:**

Dementia is associated with cognitive and functional deficits, and poses a significant personal, societal, and economic burden. Directing interventions towards older adults with self-reported cognitive complaints may provide the greatest impact on dementia incidence and prevalence. Risk factors for cognitive and functional deficits are multifactorial in nature; many are cardiovascular disease risk factors and are lifestyle-mediated. Evidence suggests that multiple-modality exercise programs can provide cognitive and functional benefits that extend beyond what can be achieved from cognitive, aerobic, or resistance training alone, and preliminary evidence suggests that novel mind-motor interventions (i.e., Square Stepping Exercise; SSE) can benefit cognition and functional fitness. Nevertheless, it remains unclear whether multiple-modality exercise combined with mind-motor interventions can benefit diverse cognitive and functional outcomes in older adults with cognitive complaints.

**Methods/Design:**

The Multiple-Modality, Mind-Motor (M4) study is a randomized controlled trial investigating the cognitive and functional impact of combined physical and cognitive training among community-dwelling adults with self-reported cognitive complaints who are 55 years of age or older. Participants are randomized to a Multiple-Modality and Mind-Motor (M4) intervention group or a Multiple-Modality (M2) comparison group. Participants exercise for 60 minutes/day, 3-days/week for 24 weeks and are assessed at baseline, 24 weeks and 52 weeks. The primary outcome is global cognitive function at 24 weeks, derived from the Cambridge Brain Sciences computerized cognitive battery. Secondary outcomes are: i) global cognitive function at 52 weeks; ii) domain-specific cognitive function at 24 and 52 weeks; iii) mobility (gait characteristics under single and dual-task conditions and balance); and 3) vascular health (blood pressure and carotid arterial measurements). We will analyze data based on an intent-to-treat approach, using mixed models for repeated measurements.

**Discussion:**

The design features of the M4 trial and the methods included to address previous limitations within cognitive and exercise research will be discussed. Results from the M4 trial will provide evidence of combined multiple-modality and cognitive training among older adults with self-reported cognitive complaints on cognitive, mobility-related and vascular outcomes.

**Trial Registration:**

ClinicalTrials.gov NCT02136368.

## Background

### Cognitive impairment in aging

With the global population aging, there is a growing urgency to identify the most effective strategies to prevent cognitive decline. Early prevention strategies may provide the greatest impact on the incidence of cognitive impairment in aging [1]. With the goal of intervening earlier, it is of interest to examine non-demented older adults with self-reported cognitive complaints, regardless of whether they have objective evidence of impairment [2]. The estimated prevalence of cognitive complaints in older adults ranges between 11 % and 56 % [3, 4]. Cognitive complaints have been associated with poorer scores on objective cognitive assessments [5], as well as cortical and hippocampal atrophy [6], and each identified cognitive complaint increases the likelihood of cognitive impairment by approximately 20 % [5].

### Relationship between cognition and vascular disease

Vascular risk factors (e.g., hypertension, obesity) are considered the most readily modifiable risk factors for dementia [7]. These risk factors, especially elevations in blood pressure (BP) and the associated arterial stiffening, reduce cerebrovascular reactivity and cerebral blood flow, and predispose older adults to greater risk of hypoperfusion in the brain [8]. Sustained hypertension and arterial stiffness are associated with a number of pathological changes in the brain [9], the occurrence of a stroke [10], the presence of neurotropic markers of Alzheimer’s disease (AD) [11], poorer scores on objective cognitive testing [12], and clinical dementia [13]. Although there is an increasing consensus on the role of vascular risk factors in cognitive impairment, few studies have investigated the effects of modifying vascular risk factors on cognitive health in either healthy older adults, or in those with cognitive impairment [14].

### Relationship between cognition and mobility

Gait dysfunction is frequently observed in older adults with cognitive impairment [15], often precedes a diagnosis of dementia [16], and has been suggested as a potentially modifiable risk factor for cognitive decline [16]. Specifically, reduced gait velocity and step length, and increased gait variability under usual (i.e., normal walking) and dual-task (DT; i.e., walking while subtracting 7 s from 100) conditions, have been associated with impaired executive functioning (EF) [17], underlying cerebrovascular disease [18], and reduced prefrontal and parietal cortical volume [19] in cognitively healthy older adults, as well as diffuse cortical atrophy [20] and abnormal neurochemical signatures within the primary motor cortex [21], among those with mild cognitive impairment (MCI). Gait dysfunction has also been associated with increased falls risk among cognitively healthy older adults [22] and those with cognitive impairment [23], as well as an increased risk for institutionalization over 5 years [24]. Interventions aimed at improving both cognition and mobility may prove most effective at reducing the risk of both cognitive and functional decline.

### Non-pharmacological interventions to prevent cognitive and functional decline

#### Exercise interventions

Healthy lifestyles, including vascular risk factor control through the habitual participation in exercise, may be an important strategy to prevent or slow the progression of AD [25]. Previous meta-analyses have revealed positive effects of aerobic exercise on cognition, with the largest effects on EF and global cognition in cognitively healthy older adults [26] and those with objective cognitive impairment [27]. Despite this evidence, a recent Cochrane review found that there is insufficient evidence to conclude that cognitive improvements are solely attributable to improved cardiovascular fitness [28]. Although more research is needed, resistance training has been found to impart cognitive benefits in older adults without cognitive impairment, including improvements in memory and EF [29], as well as frontal lobe neurophysiology [30], and elevations in circulating neural growth factors [31].

Exercise interventions aimed at improving balance and mobility have also produced discrepant findings. A Cochrane review highlighted the paucity of evidence related to the effect of exercise on mobility outcomes (i.e., usual and DT gait) and concluded that the available evidence suggesting exercise can impart moderate benefits on mobility outcomes is weak, and that further rigorously developed randomized controlled trials (RCTs) are required [32].

Multiple-modality exercise programs incorporate a number of physical exercise training types (i.e., aerobic, resistance, flexibility, and balance) [33]. Combining multiple exercise modalities may lead to greater improvements in cognition, vascular health, and functional outcomes, when compared to programs that focus on a single modality (e.g. aerobic only or resistance only programs) [34]. Previous meta-analyses in healthy older adults observed that aerobically-based, multiple-modality exercise programs can improve cognitive function, specifically EF [35] and information processing speed [36], to a greater extent than aerobic exercise alone. Results from several RCTs suggest that similar results can be expected for those self-reporting cognitive complaints [37, 38] or with objective cognitive impairment [39, 40].

Participation in three months of multiple-modality training or less has been associated with improved cognitive functioning [41], medial temporal lobe neurophysiology [42], functional mobility [41], and usual and DT gait velocity [43] in cognitively healthy older adults. Further, improved cognitive functioning has been observed in older adults with self-reported cognitive complaints [37]. Longer duration interventions might be more efficacious at improving cognition. Six months of multiple-modality exercise has been shown improve global cognition in older adults with self-reported cognitive complaints [38], as well as improve global cognition and reduce cortical atrophy in older adults with objective cognitive impairment [40], and these improvements can be maintained for up to 12 months [38]. Further, 12 months of multiple-modality exercise can improve global cognition, memory (immediate recall), and verbal fluency in older adults with objective cognitive impairment [39]. These observations suggest that multiple-modality exercise programs can serve as an effective and multifaceted approach to benefit a number of cognitive and functional outcomes in cognitively healthy older adults and in those at risk for dementia.

#### “Traditional” cognitive training & innovative cognitive-physical (“Mind-Motor”) programs

Cognitive training requires the organization and direction of a number of neurological processes, such as attention, perception, memory, and EF, and has been shown to benefit cognition in aging [44]. A recent meta-analysis revealed significant effects for cognitive training on EF, memory and global cognitive functioning, when compared to active controls (e.g., groups receiving educational DVDs or health promotion training) [45]. Further, this meta-analysis suggested significant effects for cognitive training on memory and subjective cognition functioning when compared to controls receiving no intervention [45]. Although the initial observations related to the cognitive benefits of cognitive training are promising, the improvements in cognitive functioning that are garnered following cognitive training are traditionally domain-specific [46].

Square-Stepping Exercise (SSE) is a simple, low-cost, indoor, group-based exercise program for older adults [47]. This novel program can be best described as a visuospatial working memory task with a stepping response (i.e., “mind-motor” training) and requires participants to memorize and execute progressively more complex foot placement patterns that involve forward, backward, lateral, and diagonal steps using a gridded floor mat. Although SSE was originally designed to improve lower extremity mobility in at-risk fallers [47], pilot work suggests the potential for SSE to benefit cognition [48–50]. Improvements in global cognition, attention, and mental flexibility were seen in cognitively healthy older adults after a 16-week SSE program (40 min/day, 3 days/week) [48], and improved memory and EF following a 26-week SSE program (1 day of class-based SSE/week for 50–60 min plus 10 min of daily SSE homework) [49]. Furthermore, improvements in verbal learning and memory, verbal fluency and global cognitive function were seen in older adults without dementia following a 6-month exercise plus DT training intervention (involving SSE) [50]. The available evidence regarding the effects of SSE on cognition is still preliminary and has not been examined in regard to gait dysfunction, and thus, future rigorously designed trials are required to determine the efficacy of SSE on cognitive and functional outcomes.

### Rationale and study objectives

Despite these promising observations, several limitations related to our understanding of the cognitive and functional benefits of exercise or cognitive training remain. Questions regarding the frequency, intensity, time, and type of exercise that would provide the greatest cognitive benefit are currently equivocal [51]. Other external factors, including biological sex [34] and the severity of cognitive impairment [27] also appear to modify the relationships of exercise with cognitive and physical functioning. The available evidence suggests that aerobically-based exercise programs that incorporate other exercise modalities (i.e., resistance, balance) and some form of cognitive training, might impart a significantly larger global cognitive benefit than those that focus on a single strategy [34]. Additional large-scale, rigorous RCTs are required to determine the impact of multiple-modality exercise programs combined with novel cognitive training programs, on cognition and functional mobility outcomes, and to delineate the trajectory of these improvements as well as the maintenance of training effects after follow-up, in older adults who may be at increased risk for future cognitive decline [28, 51].

The primary objective of this study is to determine whether a group-based multiple-modality exercise program combined with mind-motor training [Multiple-Modality, Mind-Motor (M4)] can lead to improved global cognitive functioning at 24 weeks, when compared to a multiple-modality exercise program alone [Multiple-Modality (M2)], among community-dwelling older adults with self-reported cognitive complaints. The study hypothesis is that improvement in global cognitive functioning will be observed in both groups; however, the improvement will be greater for M4 compared to M2. Secondary objectives include investigating whether M4 (when compared to M2) improves: i) global cognitive functioning at 52 weeks; ii) domain-specific cognitive functioning at 24 and 52 weeks; iii) mobility (gait characteristics under usual and DT conditions and balance) at 24 and 52 weeks; and iv) vascular health (BP and carotid arterial measurements) at 24 and 52 weeks.

## Methods/design

This study is a two-arm, 24-week RCT with a 28-week no-contact follow-up. Participants were randomly allocated (1:1) to either: 1) the intervention (M4) group; or 2) the comparison (M2) group. This study is being run in four waves; the first wave commenced exercise classes on 10 February 2014 and the fourth and final wave began exercise classes on 30 March 2015. All study data collection will be completed by April 2016. The design and reporting of this study follows the CONSORT (Consolidated Standards of Reporting Trials) 2010 Statement for parallel group randomized trials [52]. This RCT was registered with ClinicalTrials.gov on 29 April 2014 (Identifier: NCT02136368).

### Ethics, consent and permissions

The Western University Health Sciences Research Ethics Board approved this study (Protocol #18858 and File #102434) and all participants provided written informed consent prior to taking part in this study.

### Setting

Participants were recruited from the communities in and around Woodstock, ON, Canada. Screening visits, specific components of the measurement sessions, and the exercises classes are held at community-based locations in Woodstock. Components of the measurement sessions that could not be completed within the community take place at the Parkwood Institute in London, ON, Canada.

### Recruitment strategies

Formal recruitment commenced on 5 December 2013. Community-dwelling older adults were recruited via: 1) advertisements in the local newspapers and community partner publications; 2) posters at local businesses; 3) health fairs; 4) and word of mouth. Interested individuals contacted the study coordinator by phone, where they were provided with a brief description of the study. Individuals were then asked about their age, living status and whether they had a cognitive concern. If responses suggested study eligibility then interested individuals were invited to attend a formal in-person screening visit.

### Participants

Older adults were eligible if they: 1) were aged 55 years or older; 2) self-reported a cognitive complaint (i.e., answering “yes” to the question: “Do you feel like your memory or thinking skills have gotten worse recently?”) and; 3) had preserved instrumental activities of daily living (based on the Lawton-Brody Instrumental Activities of Daily Living scale) [53]. Exclusion criteria were: 1) probable dementia (i.e., self-reported diagnosis or Mini-Mental State Examination score < 24) [54]; 2) major depression [i.e., score ≥ 16 on the Center for Epidemiologic Studies – Depression Scale combined with clinical judgment by the Principal Investigator and study physician (R Petrella)]; 3) other neurological or psychiatric disorders; 4) recent history of severe cardiovascular conditions; 5) significant orthopaedic conditions; 6) BP unsafe for exercise (i.e., >180/100 mmHg and/or <100/60 mmHg) [55]; 7) severe sensory impairment; 8) unable to comprehend study letter of information; 9) unable to commit to at least 80 % of exercise sessions over the 24-week intervention period; and 10) any other factors that could potentially limit the ability to fully participate in the intervention.

### Interventions

#### Comparison group: Multiple-Modality (M2) exercise group

The M2 group participated in 60-minute group-based multiple-modality exercise classes, 3 days per week over 24 weeks. The class breakdown was as follows: 1) 5-minute warm-up; 2) 20 minutes of moderate-to-vigorous-intensity aerobic exercise; 3) 5-minute aerobic cool-down; 4) 10 minutes of resistance training; 5) 15 minutes of balance training, range of motion and breathing exercises; and 6) 5 minutes of stretching (see Table 1). The balance training, range of motion, and breathing exercises do not incorporate the use of additional loading (e.g., hand weights or resistance bands), and were deemed as suitable control exercises within the M2 group, as these exercises have not been found to impart cognitive benefits [56].

**Table 1:** Description of M2 and M4 interventions

M2: Multiple-Modality Exercise Group (Comparison Group)	M4: Multiple-Modality, Mind-Motor Exercise Group (Intervention Group)
Warm-up (5 minutes) o Light aerobics o Dynamic range of motion of the major joints	Warm-up (5 minutes) o Light aerobics o Dynamic range of motion of the major joints
Aerobic Exercise (20 Minutes) o Large rhythmical endurance activities (e.g., walking, marching, sequenced aerobics) o Keep HR continuously in target zone (i.e., not interval training) o Moderate to vigorous intensity o RPE: 5–8 on scale of 0–10 o Participants to check HR ½ way through and at end of aerobic exercise.	Aerobic Exercise (20 Minutes) o Large rhythmical endurance activities (e.g., walking, marching, sequenced aerobics) o Keep HR continuously in target zone (i.e., not interval training) o Moderate to vigorous intensity o RPE: 5–8 on scale of 0–10 o Participants to check HR ½ way through and at end of aerobic exercise.
Aerobic Cool Down (5 minutes) o Safely bringing heart rates down	Aerobic Cool Down (5 minutes) o Safely bringing heart rates down
Resistance Training (10 minutes) o Therabands, wall or chair exercises, core strengthening o Day 1 – Upper body focus o Day 2– Lower body focus o Day 3 – Core focus	Resistance Training (10 minutes) o Therabands, wall or chair exercises, core strengthening o Day 1 – Upper body focus o Day 2 – Lower body focus o Day 3– Core focus
Balance, Range of Motion & Breathing (15 minutes) o Keep HR *BELOW* target zone o Dynamic, static and functional balance o Breathing and relaxation exercises o Finger exercises o Range of motion (e.g., arm circles)	Mind-Motor Training (15 minutes) o Keep HR *BELOW* target zone o Progressive, group-based, Square Stepping Exercise (SSE)
Stretching (5 minutes)	Stretching (5 minutes)
TOTAL: 60 minutes 60 minutes Multiple-Modality Exercise	TOTAL: 60 minutes 45 minutes Multiple-Modality Exercise 15 minutes Mind-Motor Exercise

*Abbreviations*: *HR*, heart rate; *RPE*, rating perceived exertion

^**a**^Note: This table represents an individual session breakdown by group. Participants attended these structured 60-minute group-based exercise classes, 3 times per week for 24 weeks

#### Intervention group: Multiple-Modality and Mind-Motor (M4) exercise group

Participants in the M4 group completed a similar multiple-modality exercise class, with one exception; specifically, 15 minutes of mind-motor exercise (i.e., progressive SSE) was substituted in place of the 15 minutes of balance, range of motion and breathing exercises. This way, participants in both groups were taking part in the same amount of activity (60-minute classes; 3 days per week for 24 weeks) and were receiving the same amount of social interaction and attention from the study personnel, with the only difference being the type of activity that they received for 15 minutes during each class.

**Fig. 1:**
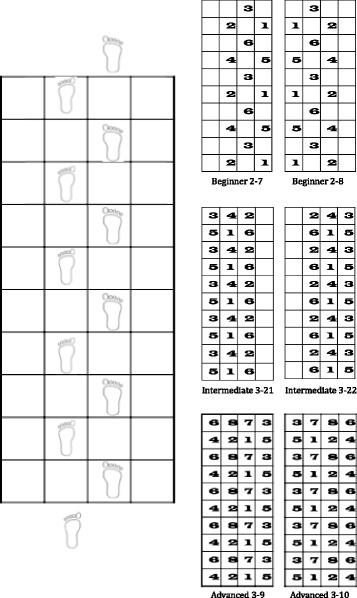
Description of Square Stepping Exercise (SSE). Participants are required to progress across a gridded floor mat while completing steps that are identical to a previously demonstrated foot placement pattern. As individuals progress, stepping pattern complexity is elevated in order to increase difficulty levels and match the individuals progressed performance capacities. Examples of beginner, intermediate and advanced patterns are shown

The SSE was selected as the mind-motor training component within this study. There are over 200 stepping patterns that range in difficulty from beginner to advanced. Participants progressed through SSE patterns each class (as a group), and started from the last successfully completed pattern performed during the previous exercise session. The goal was to progress as far as possible over the 24-week period. Participants watched an instructor demonstrate a pattern and then attempted to repeat the pattern (by memory) on the SSE mat (250 cm x 100 cm, partitioned in to 10 rows of 4 equal-sized squares (see Fig. 1). Participants worked in small groups with no more than 6 participants on an individual SSE mat. In order to promote a positive social atmosphere, participants were encouraged to assist each other during this component of the class. In order to progress to the next SSE pattern, at least 80 % of the participants had to successfully complete the pattern at least four times in a reasonable period of time. If the group did not successfully complete a specific pattern after three classes then the group would progress to the next pattern within the same difficulty level.

### Class size, compliance & intensity

Study-specific M2 and M4 exercise classes were held during morning time slots, with class sizes varying from 8 to 23 participants (depending on the wave). Attendance at exercise classes was tracked and monitored on a regular basis. The final SSE pattern completed during each session was tracked, and used as the first pattern at each subsequent training session. Participants were encouraged to attend a minimum of 80 % of classes over the course of the intervention period. During the no-contact control period, participants in both groups were encouraged to continue exercising; however, the study team did not provide the M4 group with SSE mats to continue SSE training or provide any additional intervention, and were not in contact with participants until their final study visit.

At the start of the study, each participant was provided with an individualized training heart rate (65-85 % of estimated maximum heart rate) determined via the Step Test and Exercise Prescription (STEP™) tool [57, 58]. During the aerobic exercise section, participants were encouraged to exercise at their training heart rate and/or at a rating of 5–8 on the 10-point modified Borg Rating of Perceived Exertion (RPE) scale. During either the balance/range-of-motion (M2 group) or the mind-motor (M4 group) components, participants were encouraged to work at a comfortable pace with the goal of keeping heart rates below their training heart rate. Participants were instructed to record their heart rate and RPE both immediately following the aerobic exercise component and then again following either the balance/range of motion (M2 group) or the mind-motor (M4 group) component. In order to ensure progression in aerobic training over the 24-weeks, training heart rates for each participant were recalculated at the midpoint of the intervention (i.e., 12 weeks) via the STEP™ tool.

### Instructor training

Exercise classes were led by Seniors’ Fitness Instructors, certified through the Canadian Centre for Activity and Aging [59]. Members from our research team underwent an in-person training session with one of the original developers of the SSE program and study co-investigator (R Shigematsu). Our research team then developed the SSE protocol to be used as the mind-motor component within the M4 group and conducted training with all instructors on the M2 and M4 class exercise protocols, in order to ensure standardized delivery of the programs.

### Outcome assessment

**Fig. 2:**
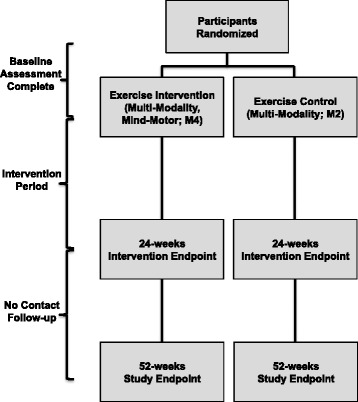
Study Flow

Outcomes are measured at baseline, 24 weeks (intervention endpoint) and 52 weeks (study endpoint) (see Fig. 2). Measurement sessions were conducted over 2 to 3 consecutive days; training emphasizes strict adherence to all written study protocols. All participants, regardless of their compliance with the exercise intervention, are telephoned one month in advance to book appointments.

#### Baseline data

Baseline measurements were obtained prior to randomization. In addition to the measurements described below, the following were also collected: demographic and general health characteristics; medical history and medications; anthropometric and fitness measurements; cognitive functioning using the Montreal Cognitive Assessment (MoCA) [60]; and current physical activity levels using the Phone-FITT physical activity interview [61].

#### Measurement protocols

##### Cognition

Global cognitive functioning, as well as domain-specific cognitive functioning, is calculated using the Cambridge Brain Sciences (CBS) computerized cognitive battery (www.cambridgebrainsciences.com). The CBS contains 12 non-verbal, culturally independent tests, which cover four broad cognitive domains (i.e., memory, reasoning, concentration, planning or EF) [62] (see Table 2). Six of the 12 tasks emphasize abstract reasoning, planning and problem solving, and these tasks were specifically included since they correlate highly with measures of general fluid intelligence [63]. The CBS tasks are fully automated, and have been used to effectively evaluate cognition in a several large-scale, population-based studies [46, 62]. The CBS cognitive battery is a computerized adaptive testing platform that randomly generates novel versions of the tasks between individual trials and can be administered in 60 minutes, thereby eliminating the potential to observe specific test-related practice effects or participant fatigue that are common to traditional paper-based cognitive assessments. The CBS tasks are conducted using laptop computers and a trackball mouse using authorized copies that have been obtained from one of the original developers and study co-investigator (A Owen). The CBS is administered on Day 1 of each measurement session for familiarization purposes only, in order to ensure participants feel comfortable using the trackball mouse and also to prevent any learning effects [62]. On Day 2 of the measurement session, the CBS testing session occurs.

**Table 2:** Description of Cambridge Brain Sciences cognitive battery

Task Name	Cognitive Domain	Brief Description	Outcome Measure
1. Monkey Ladder	Memory	Sets of numbered squares are displayed all at the same time at random locations within an invisible 5x5 grid. After a variable interval, the numbers are removed leaving just the blank squares visible. A tone cues the participant to respond by clicking on the squares in ascending numerical sequence.	Maximum level achieved
*(Visuospatial working memory)*			*The amount of squares presented increases or decreases by 1 after each trial depending on whether they responded correctly. The first trial contains 4 numbered squares.*
2. Grammatical Reasoning	Reasoning	Problems of the form “The square is not encapsulated by the circle” are displayed on the screen and the participant must indicate whether the statement correctly describes a pair of objects displayed in the centre of the screen.	Total score
*(Verbal Reasoning)*			*In order to achieve maximum points, the participant must solve as many problems as possible within the given time. The total score increases or decreases by 1 after each trial depending on whether they responded correctly.*
3. Double Trouble	Reasoning	A coloured word is displayed at the top of the screen (e.g., the word RED drawn in blue ink). Participants must indicate which of two coloured words at the bottom of the screen describes the colour that the word at the top of the screen is drawn in. The colour word mappings may be congruent, incongruent, or doubly incongruent, depending on whether or not the colours that a given word describes matches the colour that it is drawn in.	Total score
*(Colour-Word Remapping)*			*To gain maximum points, the participant must solve as many problems as possible within the given time. The total score increases or decreases by 1 after each trial depending on whether they responded correctly.*
4. Odd One Out	Reasoning	A 3x3 grid of cells is displayed on the screen. Each cell contains a variable number of copies of a coloured shape. The features that make up the objects in each cell (colour, shape, number of copies) are related to each other according to a set of rules. The participant must deduce the rules that relate the object features and select the one cell whose contents do not correspond to those rules.	Total correct
*(Deductive Reasoning)*			*To gain maximum points, the participant must solve as many problems as possible. If the response is correct, the total score increases by one point and the next problem is more complex. If the response is incorrect, the total score decreases by 1 point.*
5. Spatial Span Blocks	Memory	16 squares are displayed in a 4x4 grid. A subset of the squares flash in a random sequence at a rate of 1 flash every 900 ms. Subsequently, the mouse cursor is displayed and a tone cues the participant to repeat the sequence by clicking on the squares in the same order in which they flashed.	Maximum level achieved
*(Spatial Span)*			*To gain maximum points, the participant must solve as many problems as possible. If the response is correct, the number of illuminated squares increases by one. If the response is incorrect, the number of illuminated squares decreases by 1. The first trial contains 4 illuminated squares.*
6. Rotations	Concentration	In this variant, 2 grids of coloured squares are displayed to either side of the screen with 1 of the grids rotated by a multiple of 90°. When rotated, the grids are either identical or differ by the position of at least 1 square. In order to gain maximum points, the participant must indicate whether the grids are identical, solving as many problems as possible.	Total score
*(Spatial Rotations)*			*If the response is correct, the total score increases by the number of squares in the grid and subsequent trials have more squares. If the response is incorrect, the total score decreases by the number of squares in the grid and subsequent trials have fewer squares. The first grids contain 4 coloured squares each.*
7. Feature Match Task	Concentration	Two grids are displayed on the screen, each containing a set of abstract shapes. In half of the trials the grids differ by just one shape. In order to gain maximum points, the participant must indicate whether or not the grid contents are identical, solving as many problems as possible.	Total score
			*If the response is correct, the total score increases by the number of shapes in the grid and the number of shapes in subsequent trials increases. If the response is incorrect the total score decreases by the number of shapes in the grid and subsequent trials have fewer shapes. The first grids contain four abstract shapes each.*
8. Digit Span	Memory	Participants view a sequence of digits that appear on the screen one after another. Subsequently, participants are required to repeat the sequence of numbers by using the mouse cursor to click a series of numbered buttons that appear along the bottom of the screen.	Maximum level achieved
			*If the response is correct, the total length of the sequence increases by 1. If the response is incorrect, the total length of the sequence decreases by 1. The first trial contains a four-digit sequence.*
9. Hampshire Tree Task	Planning	Nine numbered beads are positioned on a tree shaped frame. The participant repositions the beads one-by-one so that they are configured in ascending numerical order running from left to right and top to bottom of the tree.	Total score
*(Spatial Planning)*			*After each trial, the total score is incremented by adding the minimum number of moves required × 2 (the number of moves actually made), thereby rewarding efficient planning.*
10. Paired Associates	Memory	Boxes are displayed at random locations on an invisible 5x5 grid. The boxes open one after another to reveal an enclosed object. Subsequently, the objects are displayed in random order in the centre of the grid and the participant must click on the boxes that contained them.	Maximum level achieved
			*If the response is correct, the total number of objects increases by 1. If the response is incorrect, the total number of objects decreases by 1, and subsequent trials have fewer objects. The first trial contains 4 objects.*
11. Polygons	Concentration	A pair of overlapping polygons is displayed on one side of the screen. In order to gain maximum points, the participant must indicate whether a polygon displayed on the other side of the screen is identical to one of the interlocking polygons, solving as many problems as possible.	Total score
*(Interlocking polygons)*			*If responses are correct, the total score increases by the difficulty level and the differences between the polygons becomes increasingly subtle.*
			*If the responses are incorrect, the total score decreases by the difficulty level and the difference between the polygons become more pronounced.*
12. Spatial Search	Planning	Sets of boxes are displayed on the screen in random locations within an invisible 5x5 grid. The participant must find a hidden “token” by clicking on the boxes one at a time to reveal their contents. When the token is found, it is hidden within another box.	Maximum level achieved
*(Self-Ordered Search)*			*If the response is correct, the total number of boxes increases by 1. If the response is incorrect, the total number of boxes decreases by 1, and subsequent trials have fewer boxes. The first trial contains 5 boxes.*

Note: All tasks are performed for a total of 5 minutes; three 90-second blocks, separated by two 15-second rest periods. During the rest periods, the neuropsychological tasks are hidden from view. Following each rest period, the task is returned to view, and participant continue from the last, correctly completed level of difficulty

Composite scores for each cognitive domain will be derived using previously published methods [64] as follows: i) calculating baseline group means and standard deviations from each task; ii) for each task, converting scores to standardized z scores (subtracting baseline group mean from raw score and dividing by baseline group SD); and then iii) within each domain, averaging task standardized scores to create domain-specific standardized scores. The four domain-specific standardized scores will then be averaged to create a global cognitive functioning score.

##### Ambulatory BP monitoring

Participants are fitted with a 24-hour ambulatory BP monitor (Model 90207, Spacelabs Inc., Redmond, WA, USA). A total of 40 readings are recorded over a 24-hour period, with one measurement obtained every half hour during the day (06:00–22:00), and once every hour during the night (22:00–06:00). Participants are instructed to relax the arm and remain still during cuff inflation and deflation; in case of measurement error, the monitor performs an automatic repeat attempt two minutes later. Participants are instructed to keep the cuff on for the entire 24-hour period and to abstain from showering or water activities. An activity log is provided to record any events that could affect BP, such as physical activity or stressful situations. A minimum of 32 measurements (80 %) will be required for statistical analysis. Systolic and diastolic ambulatory BP will be averaged for day-time hours, night-time hours, and then over the entire 24-hour period [65].

##### Common carotid arterial ultrasonography

Participants are instructed to avoid engaging in vigorous exercise or drink alcohol for 24 hours, avoid caffeine and smoking for 12 hours, and fast for 4 hours prior to the ultrasound measurement. Participants are instrumented with a standard three lead electrocardiogram and undergo 10 minutes of supine rest in a quiet, temperature controlled room. With the participant’s head turned approximately 45 degrees towards the left, a 10Mhz transducer is placed longitudinally along the right carotid artery, 1–2 cm proximal to the carotid sinus, to obtain two-dimensional B-mode ultrasound images (Vingmed System 5, GE Ultrasound A/S, Horton, Norway). Right common carotid arterial diameters are measured in triplicate from wall to wall, and from wall to intima media layer at end diastole and peak systole. Doppler ultrasound is used to collect pulse wave for 60 seconds. Following acquisition of the ultrasound images, carotid pulse pressure is inferred from supine brachial arterial BP (BPM-100, BPTru™ Medical Devices, Coquitlam, BC, Canada). Anatomic land marking is used to ensure that all ultrasound images are obtained from the same portions of the carotid artery and to ensure accurate comparisons over time.

Carotid arterial compliance (CAC) will be determined using the following equation: CAC = [π(D_max_/2)^2^ – π(D_min_/2)^2^]/ΔP [66], where D_max_ is the systolic carotid arterial diameter, D_min_ is the diastolic carotid arterial diameter, and ΔP is the automated supine brachial pulse pressure. Carotid intima-media thickness (IMT) will be determined by subtracting the carotid arterial lumen diameter from the arterial diameter at diastole. Two trained technicians are responsible for obtaining and analyzing all ultrasound images, with the same technician performing all assessments on a given participant whenever possible. Immediately following the ultrasound measurement, all participants are offered a standardized snack.

##### Gait

Spatiotemporal gait characteristics are collected using a portable electronic walkway system with embedded pressure sensors [GAITRite® System; 580 x 90 x .63 cm (L x W x H), an active electronic surface area 792 x 610 cm (L x W), with a total of 29,952 pressure sensors, scanning frequency of 60 Hz, Software version 4.7.1, CIR Systems, Peekskill, NY, USA]. The pressure exerted by the feet during ambulation across the mat activates the embedded pressure sensors in order to sense and digitally reconstruct the relative arrangement of footfall patterns within a two-dimensional space. The GAITRite® is a valid and reliable tool for gait analysis in healthy older adults [67] and in those with mobility impairments [68]. Participant start and endpoints are positioned 1.5 metres from either end of the mat in order to avoid the recording of acceleration and deceleration phases of the gait cycle [68].

Following the demonstration of a usual (i.e., standard gait with no DT demands) walk by the assessor, participants complete two usual walking trials at their preferred speed (i.e., a single habitualization trial followed by a single collected trial that is to be used for analysis). Participants then perform three separate DT walking conditions (counting backwards by ones; phonemic verbal fluency task; subtracting serial sevens) at a self-selected “usual” pace; for each condition, participants will complete two trials (across the mat and then back to the starting point). There will be no instruction to prioritize gait or responses to the cognitive tasks during the DT conditions. For the “counting backwards by ones” condition and the serial subtraction condition, participants are instructed to start from 100, 90, and 80 at baseline, 24 weeks and 52 weeks, respectively. For the phonemic verbal fluency condition, participants are instructed to name as many animals, vegetables, and countries, at baseline, 24 weeks, and 52 weeks respectively.

The gait performance from the two trials for each DT condition will be combined and the average performance within specific gait parameters will be used for analysis. Under both usual and DT conditions, the following will be examined: average gait velocity (cm/s), step length (cm), and stride time variability [coefficient of variation (CoV), expressed as a percentage = (SD/mean) x 100]. In recordings of the usual and DT walks, footfalls that do not entirely fall on the walkway at the start and the end will be removed prior to analyses.

##### Balance

Balance is assessed using the Fullerton Advanced Balance (FAB) Scale, a valid and reliable tool that was developed to identify emerging static and dynamic balance issues in functionally independent older adults [69]. Individual performance across 10 separate balance tasks is evaluated and scored on a Likert Scale (ranging from 0–4) following strictly defined criteria. The individual scores are summed to provide a total balance score, where higher scores reflect better balance performance (score ranges from 0 to 40).

##### Other measurements

Other measurements were taken in order to further describe the sample, help with explaining study findings, and for planned sensitivity analyses.

*The Phone-FITT:* This interview can be administered over the phone or in person, and is a valid and reliable method to evaluate both instrumental activities of daily living (household activity) and leisure time activities (recreational activity) [61]. The Phone-FITT evaluates the frequency of activities, the average duration of participation during a given bout of the activity, and the perceived intensity at which the activity was performed, during an average week in the past month. Summary scores are calculated for household, recreational and total physical activity. In addition to being measured in-person as part of the measurement sessions, the Phone-FITT was also administered via telephone each month throughout exercise intervention, in order to track other activity that participants were undertaking.

*Clinic BP.* Following 5 minutes of seated rest, BP is measured in triplicate from the brachial artery using an automated oscillometer (BPM-100, BPTru™ Medical Devices, Coquitlam, BC, Canada), with each measure separated by a 2-minute rest period. Clinic BP will be calculated as the average of the last two measurements.

*Anthropometric measurements:* Body weight (to the nearest kg) is measured with a standard weigh scale and height (to the nearest cm) is measured with a stadiometer (Health-o-Meter, Continental Scale Corp., Chicago, Il, USA). Waist circumference (to the nearest cm) is also measured following a normal exhalation at the mid-point between the twelfth rib and the upper boarder of the iliac crest [55].

*Fitness:* Predicted maximal oxygen uptake (pVO_2_max) is calculated using the STEP™ tool [57, 58]. Participants are instructed to step up and down a set of two steps (20 cm high), 20 times, at a comfortable pace. An algorithm using age, sex, time to complete the test, and post-test heart rate generates the pVO2max. This tool is also used to provide participants with a target heart rate for exercise at the start of the study and again at the mid-way point of the exercise intervention (as described previously).

### Sample size

The sample size calculation is based on the primary outcome and analysis. To our knowledge, no study to date has observed the effect of a 6-month multiple-modality and mind-motor exercise intervention on global cognitive functioning in older adults with cognitive complaints. A meta-analysis on the impact of aerobic fitness training on cognition in older adults suggested that physical exercise can improve cognition with an effect size of *d* = 0.48 [35]. Although the CBS is grounded in well-validated neuropsychological tests [46], it has not been used to date as an outcome in published exercise intervention studies. For these reasons, sample size for the proposed study must be approximated by using the effect size approach, combined with feasibility and comparisons to sample sizes used in other similar studies.

With 52 participants per group, our study would have 80 % power at the 5 % significance level to detect an effect size (mean difference divided by SD) of 0.55, a moderate effect size. We estimated a dropout rate of 20 % during the 24-week period, which increased our calculation to 65 participants per group. Thus, we proposed that 130 participants (65 participants per group) is a reasonable sample size.

The 20 % drop out rate is conservative since we observed a drop out rate of 16 % in a previous study [50]. This sample size may also be considered conservative since we will be using a variant of Analysis of Covariance (ANCOVA) to perform data analysis for the primary outcome. This proposed sample size is also in line with two exercise intervention studies for older adults with cognitive complaints; specifically, the MAX trial [37], where 126 individuals were enrolled, and a trial by Lautenschlager et al. [38], where 170 participants were enrolled.

### Randomization and allocation concealment

The randomization sequence was computer-generated (1:1 in one block of 130) and concealed using envelopes until interventions were assigned. Following baseline measurement, the Research Coordinator (who was not involved in generating the randomization sequence) enrolled and allocated participants to either the M4 or M2 group.

### Blinding

The CBS cognitive battery measurement will be blinded at 24 and 52 weeks. Additionally, wherever possible, study personnel conducting other aspects of the assessments will be blinded to group allocation. Due to the nature of the intervention, neither participants nor the exercise instructors can be blinded to group allocation. The principal investigator and investigators conducting the statistical analysis are also be blinded to group allocation.

### Statistical methods

We will analyze data based on an intent-to-treat approach, using mixed models for repeated measurements, which encompasses ANCOVA as a special case [70]. Therefore, we will include all enrolled participants in analyses and analyze data according to the randomization scheme. For our primary outcome, we will examine the difference between the M4 and M2 groups at 24 weeks in mean change of global cognitive functioning. Our secondary analysis will include examining the difference between groups at 52 weeks in mean change of global cognitive functioning. Next, we will examine differences between groups at 24 at 52 weeks on i) cognitive outcomes: memory, reasoning, concentration and EF; ii) mobility-related outcomes: usual and DT gait velocity, step length and stride time variability, and total FAB score; and iii) vascular outcomes: 24-hour systolic and diastolic BP, CAC, and carotid IMT.

For our primary outcome, we will also conduct sensitivity analyses whereby: 1) we will additionally adjust for age, sex, and baseline cardiorespiratory fitness; and 2) we will only include participants who complete a 24-week assessment and attend at least 80 % of exercise classes (i.e., “all-completers analysis”). For our primary analysis, we will also examine interactions involving age, sex, and baseline cognitive functioning (via the MoCA score). Two-sided P-values less than 0.05 will be claimed as statistically significant; however, interpretation of study results will primarily be based on estimation and associated 95 % confidence intervals [52].

## Discussion

This study will evaluate the effects of a 24-week multiple-modality plus mind-motor exercise program on global cognitive functioning, as well as domain-specific cognitive functioning, indices of cardiovascular health, and functional mobility in a sample of community-dwelling older adults with subjective cognitive complaints.

With the aging population and increased life expectancy, novel interventions aimed at preventing or slowing the onset of chronic diseases are needed. The cognitive continuum in aging suggests that strategies aimed at preventing or mitigating the progression of cognitive impairment might be most effective when targeting individuals who are within the earliest phase along the pathological cognitive continuum (i.e., prior to the establishment of objective cognitive impairment) [1]. Improving cognition or reducing the risk of cognitive decline in individuals who report cognitive concerns may help to reduce the future incidence of more serious forms of cognitive impairment [2].

Physical and cognitive activities, social engagement and vascular risk factor modification have all been suggested as important strategies for the prevention of cognitive decline [71]. Literature suggests that healthy older adults [41], as well as those with MCI [39] have improved EF following multiple-modality exercise programs. Further, initial research related to cognitive training has shown improvements in cognition, albeit domain-specific improvements in healthy older adults [46]. Recent evidence continues to suggest that cognitive training interventions produce cognitive improvements that are reserved for the cognitive domains that are actively being trained [46] and unless there is significant progression in task difficulty through the intervention, there are very little transfer effects encountered [34, 72].

By combining exercise and cognitive training programs, improvements in cognitive functioning may be additive [34]. The combination of a cognitive training paradigm within a physical exercise program may be superior to interventions that deliver these training modalities in isolation [34, 72]. The mind-motor intervention used in this study, Square-Stepping Exercise (SSE), combines physical and cognitive tasks and also promotes social engagement, which itself has been shown to benefit cognition [73]; thus, SSE might be a preferred cognitive training program for older adults compared to other available options (i.e., computerized cognitive training). Combining group-based multiple-modality exercise training with mind-motor training may provide concurrent and complementary cognitive and vascular benefits, while providing greater cognitive benefits than either intervention alone.

The vascular benefits of aerobically-based exercise training are well documented; however, the impact of aerobic exercise on cognition and brain health has yet to be unequivocally discerned [28, 34]. Further, although a number of studies have investigated the cognitive benefits of cognitive training, there remains very little evidence regarding the potential vascular benefits that can be garnered through cognitive training. By combining a multiple-modality exercise program with mind-motor training, we aim to reduce risk factor burden related to a number of chronic diseases and conditions, including cardiovascular disease, mobility limitations, and cognitive impairment and dementia, in an attempt to provide the most effective strategy to promote active and successful aging [72].

Limitations of the design and implementation of the study must also be considered. Participation was limited to a group of motivated volunteers, available during daytime hours, who are able to commit to a 24-week exercise program. In this study, we chose to use an active control group (M2 exercise program) as our comparison group rather than including an inactive control group. However, recent reviews [74] have drawn attention to the limited number of investigations on the effects of exercise in older adults with cognitive impairment that include an active control group comparison, and have recommended that future studies address this issue. The inclusion of an active control group allows for the control of other factors such as the social interaction associated with group exercise classes; however, there is evidence that implicates low intensity exercise interventions with improvements in cognition and physical function [37]. Future studies might consider also including usual-care control groups, in addition to an active comparison group in their study design. The definition of what classifies a “subjective cognitive complaint” has yet to be elucidated in the literature. We chose to use a simple question to measure whether individuals had self-reported a cognitive complaint, following the methods used by Barnes and colleagues [37]. Future studies should consult the recently published conceptual framework [75] in order to determine the most appropriate methods to evaluate subjective cognitive decline and accurately identify individuals who are at increased risk for dementia [2].

With the global population aging, there is growing urgency to identify the most effective methods to reduce cardiovascular disease risk factor burden, the establishment of functional limitations, and the development of cognitive impairment. The Multiple-Modality, Mind-Motor study has been designed to simultaneously address these concerns and determine whether a multiple-modality exercise program combined with mind-motor training can improve cognition, vascular health, and mobility in older adults with cognitive complaints, to a greater extent than multiple-modality exercise programs alone.

## References

[1] Sperling RA, Aisen PS, Beckett LA, Bennett DA, Craft S, Fagan AM (2011). Toward defining the preclinical stages of Alzheimer’s disease: recommendations from the National Institute on Aging-Alzheimer’s Association workgroups on diagnostic guidelines for Alzheimer’s disease. Alzheimers Dement..

[2] Jessen F, Wolfsgruber S, Wiese B, Bickel H, Mosch E, Kaduszkiewicz H (2014). AD dementia risk in late MCI, in early MCI, and in subjective memory impairment. Alzheimers Dement..

[3] Jorm AF, Christensen H, Korten AE, Jacomb PA, Henderson AS (2001). Memory complaints as a precursor of memory impairment in older people: a longitudinal analysis over 7–8 years. Psychol Med..

[4] Waldorff FB, Siersma V, Vogel A, Waldemar G (2012). Subjective memory complaints in general practice predicts future dementia: a 4-year follow-up study. Int J Geriatr Psychiatry..

[5] Amariglio RE, Townsend MK, Grodstein F, Sperling RA, Rentz DM (2011). Specific subjective memory complaints in older persons may indicate poor cognitive function. J Am Geriatr Soc..

[6] Saykin AJ, Wishart HA, Rabin LA, Santulli RB, Flashman LA, West JD (2006). Older adults with cognitive complaints show brain atrophy similar to that of amnestic MCI. Neurology..

[7] Yasar S, Xia J, Yao W, Furberg CD, Xue QL, Mercado CI (2013). Antihypertensive drugs decrease risk of Alzheimer disease. Ginkgo Evaluation of Memory Study. Neurology..

[8] Dai W, Lopez OL, Carmichael OT, Becker JT, Kuller LH, Gach HM (2008). Abnormal regional cerebral blood flow in cognitively normal elderly subjects with hypertension. Stroke..

[9] King KS (2014). Arterial stiffness as a potential determinant of beta-amyloid deposition. JAMA Neurol..

[10] O’Donnell MJ, Xavier D, Liu L, Zhang H, Chin SL, Rao-Melacini P (2010). Risk factors for ischaemic and intracerebral haemorrhagic stroke in 22 countries (the INTERSTROKE study): a case–control study. Lancet..

[11] Rodrigue KM, Rieck JR, Kennedy KM, Devous MDS, Diaz-Arrastia R, Park DC (2013). Risk factors for beta-amyloid deposition in healthy aging: vascular and genetic effects. JAMA Neurol..

[12] Liu H, Gao S, Hall KS, Unverzagt FW, Lane KA, Callahan CM (2013). Optimal blood pressure for cognitive function: findings from an elderly African-American cohort study. J Am Geriatr Soc..

[13] Tsao CW, Seshadri S, Beiser AS, Westwood AJ, Decarli C, Au R (2013). Relations of arterial stiffness and endothelial function to brain aging in the community. Neurology..

[14] Naqvi R, Liberman D, Rosenberg J, Alston J, Straus S (2013). Preventing cognitive decline in healthy older adults. CMAJ..

[15] Montero-Odasso M, Bergman H, Phillips NA, Wong CH, Sourial N, Chertkow H (2009). Dual-tasking and gait in people with mild cognitive impairment. The effect of working memory. BMC Geriatr..

[16] Mielke MM, Roberts RO, Savica R, Cha R, Drubach DI, Christianson T (2013). Assessing the temporal relationship between cognition and gait: slow gait predicts cognitive decline in the Mayo Clinic Study of Aging. J Gerontol A Biol Sci Med Sci..

[17] Hausdorff JM, Schweiger A, Herman T, Yogev-Seligmann G, Giladi N (2008). Dual-task decrements in gait: contributing factors among healthy older adults. J Gerontol A Biol Sci Med Sci..

[18] Rosano C, Brach J, Studenski S, Longstreth WTJ, Newman AB (2007). Gait variability is associated with subclinical brain vascular abnormalities in high-functioning older adults. Neuroepidemiology..

[19] Holtzer R, Epstein N, Mahoney JR, Izzetoglu M, Blumen HM (2014). Neuroimaging of mobility in aging: a targeted review. J Gerontol A Biol Sci Med Sci..

[20] Annweiler C, Beauchet O, Bartha R, Montero-Odasso M (2013). Slow gait in MCI is associated with ventricular enlargement: results from the Gait and Brain Study. J Neural Transm..

[21] Annweiler C, Beauchet O, Bartha R, Wells JL, Borrie MJ, Hachinski V (2013). Motor cortex and gait in mild cognitive impairment: a magnetic resonance spectroscopy and volumetric imaging study. Brain..

[22] Mirelman A, Herman T, Brozgol M, Dorfman M, Sprecher E, Schweiger A (2012). Executive function and falls in older adults: new findings from a five-year prospective study link fall risk to cognition. PLoS One..

[23] Muir SW, Speechley M, Wells J, Borrie M, Gopaul K, Montero-Odasso M (2012). Gait assessment in mild cognitive impairment and Alzheimer’s disease: the effect of dual-task challenges across the cognitive spectrum. Gait Posture..

[24] Verghese J, LeValley A, Hall CB, Katz MJ, Ambrose AF, Lipton RB (2006). Epidemiology of gait disorders in community-residing older adults. J Am Geriatr Soc..

[25] Smetanin P, Kobak P, Briante C, Stiff D, Sherman G, Ahmad S. Rising Tide: the impact of dementia on Canadian society. 2010. http://www.alzheimer.ca/~/media/Files/national/Advocacy/ASC_Rising_Tide_Full_Report_e.pdf. Accessed 12 Jan 2013

[26] Voss MW, Nagamatsu LS, Liu-Ambrose T, Kramer AF (2011). Exercise, brain, and cognition across the life span. J Appl Physiol..

[27] Gates N, Fiatrone Singh MA, Sachdev PS, Valenzuela M (2013). The effect of exercise training on cognitive function in older adults with mild cognitive impairment: a meta-analysis of randomized controlled trials. Am J Geriatr Psychiatry..

[28] Young J, Angevaren M, Rusted J, Tabet N (2015). Aerobic exercise to improve cognitive function in older people without known cognitive impairment. Cochrane Database Syst Rev..

[29] Liu-Ambrose T, Nagamatsu LS, Graf P, Beattie BL, Ashe MC, Handy TC (2010). Resistance training and executive functions: a 12-month randomized controlled trial. Arch Intern Med..

[30] Nagamatsu LS, Handy TC, Hsu CL, Voss M, Liu-Ambrose T (2012). Resistance training promotes cognitive and functional brain plasticity in seniors with probable mild cognitive impairment. Arch Intern Med..

[31] Cassilhas RC, Viana VA, Grassmann V, Santos RT, Santos RF, Tufik S (2007). The impact of resistance exercise on the cognitive function of the elderly. Med Sci Sports Exerc..

[32] Howe TE, Rochester L, Neil F, Skelton DA, Ballinger C. Exercise for improving balance in older people (Review). The Cochrane Library. 2011;9(11):CD004963. 10.1002/14651858.CD004963.pub3 PMC11493176 22071817

[33] Voelcker-Rehage C, Godde B, Staudinger UM (2010). Physical and motor fitness are both related to cognition in old age. Eur J Neurosci..

[34] Gregory MA, Gill DP, Petrella RJ (2013). Brain health and exercise in older adults. Curr Sports Med Rep..

[35] Colcombe SJ, Kramer AF (2003). Fitness effects on the cognitive function of older adults: A meta-analytic study. Psychol Sci..

[36] Smith PJ, Blumenthal JA, Hoffman BM, Cooper H, Strauman TA, Welsh-Bohmer K (2010). Aerobic exercise and neurocognitive performance: a meta-analytic review of randomized controlled trials. Psychosom Med..

[37] Barnes DE, Santos-Modesitt W, Poelke G, Kramer AF, Castro C, Middleton LE (2013). The Mental Activity and eXercise (MAX) trial: a randomized controlled trial to enhance cognitive function in older adults. JAMA Intern Med..

[38] Lautenschlager NT, Cox KL, Flicker L, Foster JK, van Bockxmeer FM, Xiao J (2008). Effect of physical activity on cognitive function in older adults at risk for Alzheimer disease: a randomized trial. JAMA..

[39] Suzuki T, Shimada H, Makizako H, Doi T, Yaffe K (2012). Effects of multicomponent exercise on cognitive function in older adults with amnestic mild cognitive impairment: a randomized controlled trial. BMC Neurology..

[40] Suzuki T, Shimada H, Makizako H, Doi T, Yoshida D, Ito K (2013). A randomized controlled trial of multicomponent exercise in older adults with mild cognitive impairment. PLoS One..

[41] Forte R, Boreham CA, Leite JC, De Vito G, Brennan L, Gibney ER (2013). Enhancing cognitive functioning in the elderly: multicomponent vs. resistance training. Clin Interv Aging.

[42] Li R, Zhu X, Yin S, Niu Y, Zheng Z, Huang X (2014). Multimodal intervention in older adults improves resting-state functional connectivity between the medial prefrontal cortex and medial temporal lobe. Front Aging Neurosci..

[43] Agmon M, Kelly VE, Logsdon RG, Nguyen H, Belza B (2015). The effects of EnhanceFitness (EF) training on dual-task walking in older adults. J Appl Gerontol..

[44] Kramer AF, Bherer L, Colcombe SJ, Dong W, Greenough WT (2004). Environmental influences on cognitive and brain plasticity during aging. J Gerontol A Biol Sci Med Sci..

[45] Kelly ME, Loughrey D, Lawlor BA, Robertson IH, Walsh C, Brennan S (2014). The impact of cognitive training and mental stimulation on cognitive and everyday functioning of healthy older adults: A systematic review and meta-analysis. Ageing Res Rev..

[46] Owen AM, Hampshire A, Grahn JA, Stenton R, Dajani S, Burns AS (2010). Putting brain training to the test. Nature..

[47] Shigematsu R, Okura T, Sakai T, Rantanen T (2008). Square-stepping exercise versus strength and balance training for fall risk factors. Aging Clin Exp Res..

[48] Teixeira CVL, Gobbi S, Pereira JR, Vital TM, Hernandéz SSS, Shigematsu R (2013). Effects of square-stepping exercise on cognitive functions of older people. Psychogeriatrics..

[49] Shigematsu R (2014). Effects of Exercise Program requiring attention, memory and imitation on cognitive function in elderly persons: a non-randomized pilot study. J Gerontol Geriatric Res..

[50] Gill DP, Gregory MA, Zou GY, Shigematsu R, Hachinski V, Fitzgerald C, et al. The Healthy Mind, Healthy Mobility Trial: a novel exercise program for older adults. Med Sci Sports Exerc. 2015; doi:0.1249/MSS.0000000000000758 10.1249/MSS.0000000000000758 26285025

[51] Kelly ME, Loughrey D, Lawlor BA, Robertson IH, Walsh C, Brennan S (2014). The impact of exercise on the cognitive functioning of healthy older adults: a systematic review and meta-analysis. Ageing Res Rev..

[52] Schulz KF, Altman DG, Moher D, Group TCONSORT (2010). CONSORT 2010 Statement: updated guidelines for reporting parallel group randomised trials. BMC medicine..

[53] Lawton MP, Brody EM (1969). Assessment of older people: self-maintaining and instrumental activities of daily living. Gerontologist..

[54] Folstein MF, Folstein SE, McHugh PR (1975). “Mini-mental state”. A practical method for grading the cognitive state of patients for the clinician. J Psychiatr Res.

[55] Thompson WR, Gordon NF, Pescatello LS (2010). American College of Sports Medicines Guidelines for Exercise Testing and Prescription.

[56] Colcombe SJ, Kramer AF, Erickson KI, Scalf P, McAuley E, Cohen NJ (2004). Cardiovascular fitness, cortical plasticity, and aging. Proc Natl Acad Sci USA.

[57] Knight E, Stuckey MI, Petrella RJ (2014). Validation of the step test and exercise prescription tool for adults. Can J Diabetes..

[58] Petrella RJ, Koval JJ, Cunningham DA, Paterson DH (2001). A self-paced step test to predict aerobic fitness in older adults in the primary care clinic. J Am Geriatr Soc.

[59] Canadian Centre for Activity and Aging: Senior’s Fitness Instructors Course. http://www.uwo.ca/ccaa/training/courses/sfic/index.html. Accessed 21 Sept 2015.

[60] Nasreddine ZS, Phillips NA, Bedirian V, Charbonneau S, Whitehead V, Collin I (2005). The Montreal Cognitive Assessment, MoCA: a brief screening tool for mild cognitive impairment. J Am Geriatr Soc..

[61] Gill DP, Jones GR, Zou GY, Speechley M (2008). The Phone-FITT: a brief physical activity interview for older adults. J Aging Phys Act..

[62] Hampshire A, Highfield RR, Parkin BL, Owen AM (2012). Fractionating human intelligence. Neuron..

[63] Gray JR, Chabris CF, Braver TS (2003). Neural mechanisms of general fluid intelligence. Nat Neurosci..

[64] Monsell SE, Liu D, Weintraub S, Kukull WA (2012). Comparing measures of decline to dementia in amnestic MCI subjects in the National Alzheimer’s Coordinating Centre (NACC) uniform data set. Int Psychogeriatr..

[65] Pickering TG, Hall JE, Appel LJ, Falkner BE, Graves J, Hill MN (2005). Recommendations for blood pressure measurement in humans and experimental animals: part 1: blood pressure measurement in humans: a statement for professionals from the Subcommittee of Professional and Public Education of the American Heart Association Council on High Blood Pressure Research. Circulation..

[66] Sugawara J, Inoue H, Hayashi K, Yokoi T, Kono I (2004). Effect of low-intensity aerobic exercise training on arterial compliance in postmenopausal women. Hypertens Res..

[67] Bilney B, Morris M, Webster K (2003). Concurrent related validity of the GAITRite walkway system for quantification of the spatial and temporal parameters of gait. Gait Posture..

[68] Montero-Odasso M, Casas A, Hansen KT, Bilski P, Gutmanis I, Wells JL (2009). Quantitative gait analysis under dual-task in older people with mild cognitive impairment: a reliability study. J Neuroeng Rehabil..

[69] Rose DJ, Lucchese N, Wiersma LD (2006). Development of a multidimensional balance scale for use with functionally independent older adults. Arch Phys Med Rehabil..

[70] Fitzmaurice GM, Laird NM, Ware JH, Balding DJ, Cressie NAC, Fisher NI, Johnstone IM, Kadane JB, Molenberghs G (2011). Modelling the mean: analyzing response profiles. Applied Longitudinal Analysis.

[71] Lancet Neurology (2012). A grand plan for Alzheimer’s disease and related dementias. Lancet Neurol.

[72] Hughes TF, Becker JT, Lee CW, Chang CC, Ganguli M. Independent and combined effects of cognitive and physical activity on incident MCI. Alzheimers Dement. 2015;11(11):1377-84. 10.1016/j.jalz.2014.11.007 PMC4536189 25684687

[73] Saczynski JS, Pfeifer LA, Masaki K, Korf ES, Laurin D, White L (2006). The effect of social engagement on incident dementia: the Honolulu-Asia Aging Study. Am J Epidemiol..

[74] Law LL, Barnett F, Yau MK, Gray MA (2014). Effects of combined cognitive and exercise interventions on cognition in older adults with and without cognitive impairment: A Systematic Review. Ageing Res Rev..

[75] Jessen F, Amariglio RE, van Boxtel M, Breteler M, Ceccaldi M, Chetelat G (2014). A conceptual framework for research on subjective cognitive decline in preclinical Alzheimer’s disease. Alzheimers Dement..

